# Bridging human and animal personality: A behavioral test to assess reward sensitivity

**DOI:** 10.1016/j.isci.2025.113487

**Published:** 2025-09-02

**Authors:** Susana C.M. Ferreira, Fabiana De Angelis, Sarah Ambruosi, Giulia Ferroni, Matteo Chincarini, Charlotte Goursot

**Affiliations:** 1 Department of Interdisciplinary Life Sciences, Research Institute of Wildlife Ecology, University of Veterinary Medicine, Vienna, Austria; 2 Institute of Animal Welfare Science, University of Veterinary Medicine, Veterinärplatz 1, Wien, 1210 Vienna, Austria; 3 Institute of Livestock Sciences, Department of Agricultural Sciences, BOKU University, Gregor-Mendel-Straße 33, 1180 Vienna, Austria; 4 Epidemiology, Health and Welfare Unit, Ploufragan-Plouzané-Niort Laboratory, French Agency for Food, Environmental and Occupational Health & Safety (ANSES), BP 53, 41 rue de Beaucemaine, 22440 Ploufragan, France; 5 Department of Veterinary Medicine, University of Teramo, SP18, 64100 Teramo, TE, Italy; 6 Research Institute for Farm Animal Biology (FBN), Working Group Animal Behaviour & Welfare, Wilhelm-Stahl-Allee 2, 18196 Dummerstorf, Germany; 7 Center for Proper Housing of Ruminants and Pigs, Federal Food Safety and Veterinary Office, Route de la Tioleyre 4, 1725 Posieux, Switzerland

**Keywords:** Animal science, Social sciences, Psychology

## Abstract

Accounting for individual differences in depression or resilience is crucial to ensure individualized well-being. Differences in tendencies to approach rewards and avoid threats reflect personality and help to understand what animals want and like. At the neural level, these tendencies involve the behavioral activation system (BAS) and the behavioral inhibition system (BIS). We developed a test to investigate reactions to rewards and approach-avoidance conflicts using the domestic pig as a model. After testing 101 piglets, we showed that this test is reproducible, highly repeatable, and linked with classic personality dimensions assessed with four established personality tests. We report on individual tendencies related to approach-avoidance conflicts (BIS) and reward responsiveness (BAS), which are connected to specific personality traits. These findings address the gap between human and non-human animal personality research. Our study explores individual differences in perceived rewards, which have strong implications in promoting positive animal welfare and mental health.

## Highlights

• Behavioral inhibition vs activation systems (BIS/BAS) are linked to personality
• We present the first test measuring BIS/BAS scales in non-human animals, the BIBAGO
• The BIBAGO is highly repeatable, reproducible, and linked to personality tests
• The BIBAGO enables characterizing individuals based on their reward sensitivity

## Graphical abstract

**Figure:**
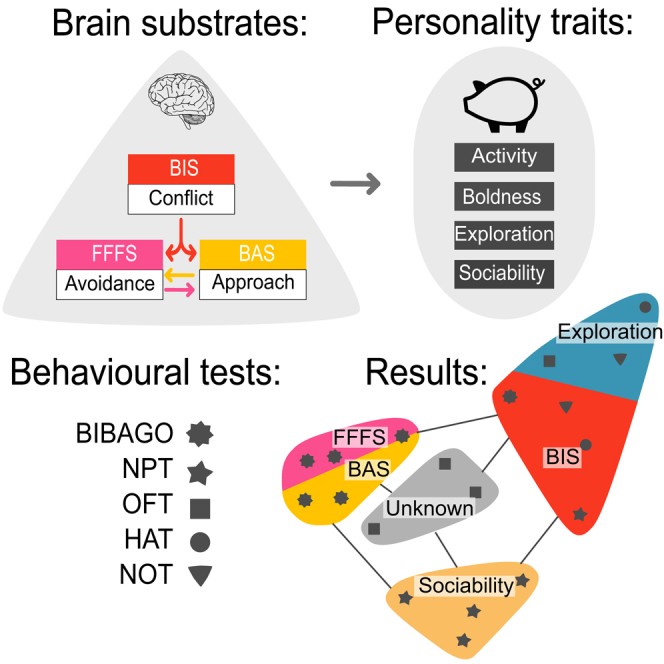

## Introduction

**Figure 1:**
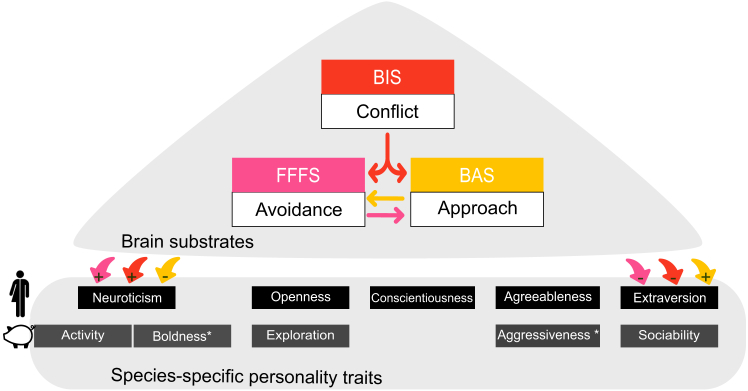
Brain motivational systems regulate approach-avoidance behaviors and underlie personality traits The non-human traits are represented above their human equivalents. The behavioral activation system (BAS) motivates the approach of rewards, and the fight-flight-freeze system (FFFS) motivates the avoidance of threats. The behavioral inhibition system (BIS) is activated during approach-avoidance conflicts, such as ambiguous and/or novel contexts. In humans, BIS is positively correlated with neuroticism^16^^,^^17^^,^^18^^,^^19^ and negatively correlated with extraversion^17^^,^^18^ (in red). Similar links have been documented regarding human FFFS^16^ (in pink). Human BAS is more diverse than BIS or FFFS as it usually includes several subscales that can reflect opposite motivations (e.g., impulsivity vs. goal drive persistence, not represented on this figure). However all BAS subscales have been documented to positively correlate with extraversion^16^^,^^18^^,^^19^ and to negatively correlate with neuroticism^16^ (in yellow). ∗In non-human animals, boldness represents the opposite of human neuroticism, and aggressiveness the opposite of human agreeableness.^1^^,^^2^ The equivalent of sociability is human extraversion, and there is currently no established equivalent of human conscientiousness.^1^^,^^2^

Everyone reacts and perceives one’s environment in an individual way. These individual behaviors are typically described along multiple personality traits or dimensions, such as the big five (Figure 1),^1^^,^^2^^,^^3^ but see Koski.^4^ Personality, in both human and non-human animals, describes consistent individual differences in behavior across time and contexts.^2^^,^^5^ These traits can inform individualized approaches in various contexts, including mental health^6^ and animal welfare^7^ programs. Certain personality traits are strongly associated to psychopathology^8^; for instance, neuroticism reflects a tendency toward the experience of negative emotions,^9^^,^^10^ which is a risk factor for depression,^11^^,^^12^^,^^13^ and extraversion is associated with experiencing more positive emotions, influencing well-being. While traditional personality tests are valuable tools for assessing individual differences in behavior, they disproportionately focus on fear reactions.^14^ This limits our understanding of individual variation within non-human animals for experiencing positive mental states that in turn is crucial for promoting positive animal welfare.^15^

The reinforcement sensitivity theory of personality is the most influential model explaining the brain mechanisms underlying human personality traits.^16^^,^^20^^,^^21^ This theory proposes three motivational systems that regulate approach-avoidance behaviors^22^: the behavioral activation system (BAS; reward-driven approach), the fight-flight-freeze system (FFFS; fear-driven avoidance), and the behavioral inhibition system (BIS; mediating approach-avoidance conflict). Brain structures involved in these systems have been documented, such as the orbitofrontal cortex (BIS), amygdala (FFFS), or nucleus accumbens (BAS).^20^ Human BIS/BAS scales, derived from self-reports,^16^^,^^23^ partly explain personality traits.^16^^,^^17^^,^^18^ High BAS is associated with extraversion, while high BIS is associated with neuroticism.^22^^,^^24^ Expanding our knowledge on the core of approach-avoidance motivations to other animals would contribute to understanding the evolutionary origins of personality and individualizing animal welfare practices.^25^ Yet, self-reports are not suitable for non-human animals, and to date no behavioral test has been developed to measure animal BIS/BAS traits.

This study developed the first behavioral test to measure motivational traits in the domestic pig (*Sus scrofa*) and examined their relationship with established personality traits. Pigs are ideal for this purpose due to their neurobiological similarity to humans.^26^ This species, highly relevant to animal welfare, also fulfills the prerequisite of showing multiple personality traits,^27^^,^^28^ resembling human personality traits. Indeed, although personality assessments in human vs. non-human animals qualitatively differ, research on non-human animal personality has been established in the last decades by documenting a similar five-traits model,^1^^,^^2^ where for instance (non-human) sociability seems to resemble (human) extraversion while (non-human) boldness and activity seem to be integrated into (human) neuroticism^1^^,^^7^(see more details in Figure 1). A major criterion for a suitable personality test is to ensure the consistency of reactions across time during this test. Per definition, repeatability of a personality test must be warranted to enable the extraction of certain personality traits.^25^^,^^29^ We (1) developed the “BIBAGO” (BIS/BAS, Goursot), a behavioral test measuring reactions to simultaneous positive (a treat ball) and negative (a moving plastic bag) stimuli in a novel context, designed to separately activate the BAS (positive stimulus), FFFS (negative stimulus), and BIS (conflict between approaching or avoiding the stimuli^22^). Moving a plastic bag has previously been validated as a negative stimulus that triggers a startle or avoidance reaction.^30^^,^^31^^,^^32^ We then (2) compared the consistency of the behaviors at different time points observed during the BIBAGO and of those observed during established personality tests^28^^,^^33^: the open-field test (OFT), the novel object test (NOT), and the human approach test (HAT), but also the novel peer test (NPT), recently developed to measure sociability in pigs (Table 1).^34^ Finally, we (3) analyzed the associations between behaviors reflecting motivational systems and personality traits. We expected greater repeatability for the BIBAGO, reflecting core motivational tendencies at the origin of the expression of personality. We also expected BAS to positively correlate with sociability and BIS to negatively correlate with boldness, consistent with the previously described links between BAS and extraversion and BIS and neuroticism^16^^,^^18^ (see Figure 1).

**Table 1:** Overview of the behaviors measured for each personality trait and by each test: OFT, NOT, HAT, BIBAGO, and NPT

Name	Test	Hypothesized personality trait(s)	Definition	Used or recommended by	Behavior as part of a dimension	Repeatable
Arena exploration (D)	OFT^a^	**exploration**/boldness	manipulating the floor or walls with the snout for at least 2 s	Finkemeier et al.^7^; O’Malley et al.^28^; Goursot et al.^33^; Leliveld et al.^35^	for boldness^36^ pharmacological validation (for boldness^37^)	yes^38^ no^37^
Locomotion (D)	OFT^a^	**activity**/proactivity/boldness	moving with at least 3 feet	Finkemeier et al.^7^; O’Malley et al.^28^; Goursot et al.^33^; Leliveld et al.^35^	for activity^39^^,^^40^^,^^41^ for boldness^36^ pharmacological validation (for boldness^37^)	yes^38^ no^37^
Vocalizations (N)	OFT^a^/BIBAGO^a^	**sociability**/proactivity/boldness/BIS/**FFFS**	emitted vocalizations	for sociability^33^^,^^42^^,^^43^ for boldness for proactivity^44^	for sociability^42^ pharmacological validation (for boldness:^37^)	no^37^ yes for proactivity^38^ to some extent for proactivity^45^
Jumping (N)	OFT^a^/HAT	**boldness/**proactivity	raising at least two legs against the wall	Finkemeier et al.^7^; O’Malley et al.^28^; Goursot et al.^33^; Leliveld et al.^35^; Reimert et al.^46^; Zebunke et al.^47^	pharmacological validation^48^	tested in this study
Object exploration (D)	NOT^a^	**exploration**/boldness	touching the novel object with the snout	Finkemeier et al.^7^; O’Malley et al.^28^; Goursot et al.^33^; Leliveld et al.^35^	for exploration^49^^,^^50^ for boldness^36^ pharmacological validation (for fearfulness^37^)	yes^50^ no^38^^,^^51^
Object exploration (N)	NOT	**exploration/**boldness		Finkemeier et al.^7^; O’Malley et al.^28^; Goursot et al.^33^	for boldness^36^	to some extent for proactivity^45^
Object exploration (L)	NOT	**boldness**/exploration		O’Malley et al.^28^; Goursot et al.^33^; Leliveld et al.^35^	for exploration^50^	yes^38^^,^^39^^,^^50^^,^^52^^,^^53^^,^^54^ no^51^ to some extent for proactivity^45^
Human exploration (L)	HAT^a^	exploration/**boldness**	touching the human with the snout	O’Malley et al.^28^; Goursot et al.^33^; Leliveld et al.^35^	unknown	yes^39^^,^^52^^,^^54^ no^55^^,^^56^
Human exploration (D)	HAT	**exploration**/boldness		Finkemeier et al.^7^; O’Malley et al.^28^; Goursot et al.^33^	for exploration^49^ for boldness:^36^	tested in this study
Tail wagging (N)	HAT^a^/NPT^a^	sociability/**BAS**	moving the tail in a consistent pattern from side to side or sudden sideways motion. A new occurrence of tail wagging was scored if the wagging was sustained for more than 3 s.	not tested in the personality context, behavior assumed to reflect positive affective states^57^^,^^58^^,^^59^^,^^60^	unknown	tested in this study
Interruption of vocalizations (D)	BIBAGO^a^/NOT^a^	boldness/**BIS**	interrupting the emission of vocalizations directly after the minute of habituation while the stimuli are introduced	not tested in the personality context, behavior that has been associated with an increased attentional state^31^^,^^61^	unknown	tested in this study
Chewing (D)	BIBAGO^a^	BAS	sound of chewing; at least two chewing sounds in a row.	this study	unknown	tested in this study
Rewards eaten (N, max 10)	BIBAGO^a^	BAS	number of chocolate raisins eaten	this study	unknown	tested in this study
Interactions with reward (D)	BIBAGO^a^	BAS	manipulating the treat ball with the snout	this study	unknown	tested in this study
Interactions with reward (L)	BIBAGO	**BIS/**BAS		this study	unknown	tested in this study
Freezing (D)	BIBAGO^a^	BIS/**FFFS**	no vocalizations emitted and no movement by any body part for 3 s or longer	not tested in the personality context, behavior reflecting an attentional state in negative contexts^62^ Because this behavior is not well documented in pigs, we cannot assume that it is comparable to freezing in rodents reflecting FFFS.	unknown	tested in this study
Back of the pen (D)	NPT^a^	sociability	being at the back of the pen	this study	unknown	tested in this study
Climbing the fence (N)	NPT	sociability	raising at least two legs against the fence	this study	unknown	tested in this study
Front of the pen (N)	NPT	sociability	being at the fence area of the pen	this study	unknown	tested in this study
Front of the pen (L)	NPT	sociability		this study	unknown	tested in this study
Middle of the pen (D)	NPT^a^	sociability	being at the middle of the pen	this study	unknown	tested in this study
Sudden display (D)	NPT^a^	**sociability/**BAS	isolated, sporadic movement such as hoping, scampering, pivoting or head tossing	not tested in the personality context, potential similarities with play behavior^34^^,^^59^^,^^63^	unknown	tested in this study
Turning back (N)	NPT^a^	sociability	turning the head back to the fence while body facing the back of the pen	this study	unknown	tested in this study
Walking by the fence (D)	NPT^a^	sociability	moving with at least 3 feet in one direction alongside the fence and then 3 feet in the opposite direction	Ambruosi et al.^34^	for sociability^34^	yes^34^
Nose-nose interactions (L)	NPT	sociability	touching the snout of the novel pig with the snout (s)	this study	unknown	tested in this study
Nose-nose interactions (N)	NPT	sociability		Ambruosi et al.^34^	for sociability^34^^,^^42^	no^34^

OFT, open-field test; NOT, novel object test; HAT, human approach test; BIBAGO, BIS/BAS test; NPT, novel peer test; D, duration in seconds; N, number of occurrences; L, latency in seconds; BIS, behavioral inhibition; BAS, behavioral activation; FFFS, fight-flight-freeze systems. We document how each behavioral variable has been assigned to the personality trait in previous works, according to the definitions of Réale et al.^2^ that have been adapted to farm animals.^7^ Behaviors can be assigned to multiple personality traits according to the studies (references are given). In the column hypothesized personality trait(s), we highlighted in bold the trait to which we assigned each behavior in this study. For example, most variables measured during the OFT and NOT can reflect both boldness and exploration, according to the study rationale. This is due to a lack of standardization at the design level and lack of statistical robustness between personality studies within a species (e.g., here pigs).

a Represent variables included in the network analysis and extended exploratory factor analysis in this study.

## Results

### OFT has the lowest repeatability across time

We analyzed 5 different tests with a total of 26 different behavioral variables hypothesized to measure personality dimensions, as continuous traits. First, we assessed the overall repeatability of each test. The BIBAGO test, developed in this study and designed to separately activate the BAS and BIS, showed the highest overall repeatability (distance-based intraclass correlation coefficients: dICC = 0.355 ± 0.058), and this finding was confirmed in an independent subset of animals (“BIBAGO2,” see STAR Methods section, dICC = 0.433 ± 0.097). The NPT had the next highest repeatability (dICC = 0.272 ± 0.053), followed by NOT (dICC = 0.253 ± 0.089) and HAT (dICC = 0.153 ± 0.061). OFT had poor repeatability (dICC = −0.011 ± 0.048). After accounting for mother identity and replicate (batch) effects, the time point of testing explained over 20% of the variation in behaviors measured within OFT (permutational multivariate analysis of variance test marginal R^2^ = 0.237, *p* = 0.001) while for the BIBAGO1 and 2, NPT, HAT, and NOT, the time point explained less than 10% of the behavior variation (Table S3). This suggests that OFT behavior changed more substantially on the second testing time point than behaviors in other tests.

The repeatability of each measured behavior was accessed individually. Thirteen behaviors showed poor repeatability across the two testing time points (Table S4) and were therefore excluded from further analysis, with three exceptions: locomotion, vocalizations, and jumping, all measured during the OFT (see STAR Methods section for justification).

### Motivational systems are linked to personality traits

**Figure 2:**
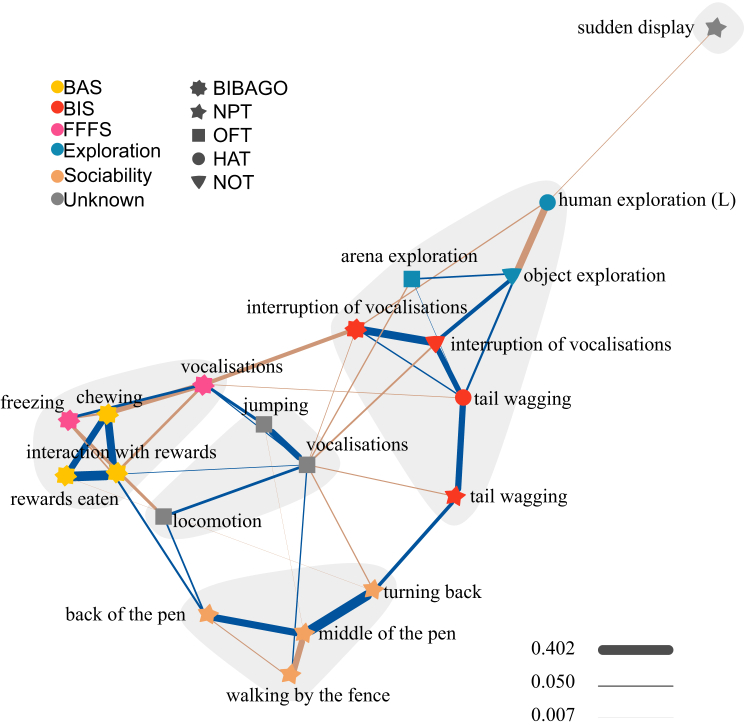
Bridging personality traits and BIS/BAS Gaussian graphical model for behaviors measured within 5 different tests hypothesized to measure personality traits and BIS/BAS tendencies. Blue edges represent positive and brown edges represent negative correlations. Edge thickness represents the strength of the association ranging from −0.286 to 0.402. Gray shaded areas represent the clusters of behaviors. Shapes of the nodes represent the test, and colors the hypothesized personality trait. See Table 1 for description of behaviors. *n* = 80. BIS, behavioral inhibition; BAS, behavioral activation; FFFS, fight-flight-freeze systems; OFT, open-field test; NOT, novel object test; HAT, human approach test; BIBAGO, BIS/BAS test; NPT, novel peer test; L, latency

We analyzed the associations between behaviors across the different tests measuring the motivational systems (BAS, BIS, and/or FFFS) and personality traits (exploration, sociability, boldness, and activity; see Table 1). The network included 20 behaviors (nodes) and 42 edges (Figure 2) and represents inter-individual behavioral differences. Network centrality values and stability of network structure are depicted in Figure S2. Given the exploratory nature of this approach, and the inconsistencies in the literature attributing different personality traits for the same behaviors (Table 1), we applied a cluster analysis and re-assigned behavioral traits to specific behaviors (Figure 2).

Our initial hypothesis was that tail wagging reflected BAS activity; however, it clustered with behaviors hypothesized to reflect BIS (interruption of vocalizations, in red, see Figure 2). Therefore, we reclassified tail wagging as BIS-related behavior (also in red, see Figure 2). These BIS-related behaviors are clustered with behaviors reflecting the exploration trait (arena and object exploration, in blue; see Figure 2). Human exploration (latency) has been described as reflecting exploration^54^ but also boldness^33^ in pigs. Our results show a strong link of human exploration with object exploration; thus, we re-assigned its trait from boldness to exploration (in blue, see Figure 2).

Behaviors hypothesized to measure BAS (chewing, interactions with reward, and rewards eaten, in yellow; see Figure 2) formed a cluster together that included behaviors for which the BIS/FFFS classification was unclear (freezing and vocalizations measured within BIBAGO). Given the negative but close links between these unclear behaviors and BAS-related behaviors (reflecting approach motivations), we concluded that they were more likely to reflect FFFS (avoidance motivations, the opposite of approach motivations, in pink; see Figure 2), rather than BIS supposedly more independent from BAS.^23^

Jumping, locomotion, and vocalizations measured during the OFT formed a cluster together. While these could represent boldness, i.e., the common denominator for these behaviors (Table 1), their lack of repeatability suggests they may not reflect personality traits.^29^ Therefore, we designated these OFT behaviors as belonging to an “unknown” category, rather than to a personality trait per se (in gray, see Figure 2).

The remaining behaviors hypothesized to reflect sociability (back and middle of the pen, walking by the fence, and turn back, in orange; see Figure 2) cluster together, apart from sudden display, which remained isolated at the periphery of the network. Consequently, we re-assigned the sudden display to an “unknown” trait.

**Figure 3:**
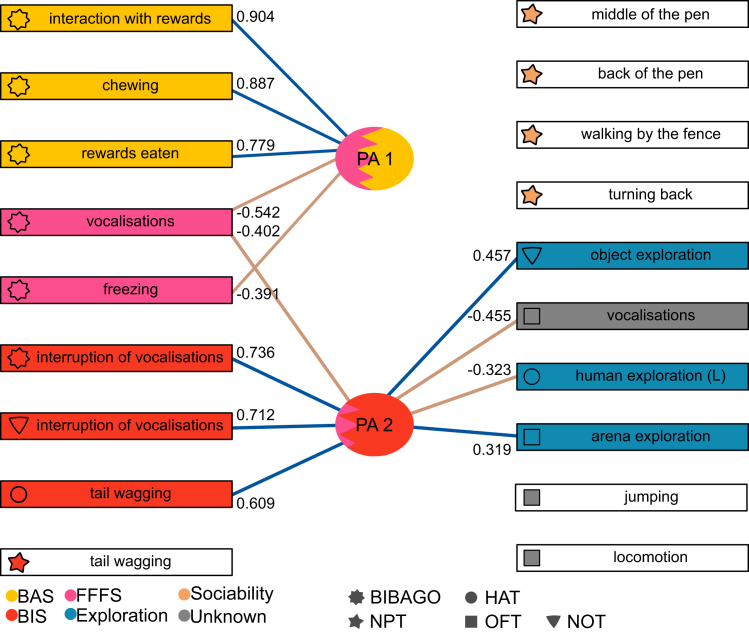
Coefficients (loadings) of extended factor analysis from behaviors across multiple tests Factor loadings of <0.300 have not been included. Shapes of the nodes represent the test, and colors the hypothesized personality trait. BIS, behavioral inhibition; BAS, behavioral activation; FFFS, fight-flight-freeze systems; OFT, open-field test; NOT, novel object test; HAT, human approach test; BIBAGO, BIS/BAS test; NPT, novel peer test; L, latency. See Table 1 for description of behaviors and initial hypothesis.

Extended exploratory factor analysis (EFA) largely supported the network results. Sudden display was excluded from the EFA due to a low measure of sampling adequacy (MSA = 0.38). The remaining variables were suitable for EFA (overall MSA = 0.64, Bartlett’s test ꭓ^2^ = 602.768, degrees of freedom = 171, *p* < 0.001). Initially, all behaviors hypothesized to correspond to BIS/BAS/FFS were included, and 2 factors were found (Table S5). BAS behaviors and FFFS behaviors loaded on a first principal axis (PA1), while BIS behaviors with tail wagging (NPT) loaded on a second principal axis (PA2, see Figure 3 and Table S5. FFFS behavior vocalizations also loaded on the PA2. All remaining behaviors hypothesized to measure sociability and exploration and the unknown categories were associated with the resulting PA1 and PA2 (Table S6). Behaviors hypothesized to measure exploration loaded onto PA2, along with OFT vocalizations, reflecting the unknown category (Table S6 and Figure 3).

## Discussion

Approach-avoidance conflicts are central to everyday decision-making. Understanding the origins of individual differences in resolving these conflicts is crucial for promoting well-being and treating mental health disorders like anxiety, depression, and addiction. We have developed and applied a new test to measure approach-avoidance tendencies and have expanded current personality knowledge in non-human animals to new and positive aspects of personality that traditional tests do not capture. This test identifies behaviors reflecting approach tendencies (BAS) that are distinct from behaviors reflecting avoidance tendencies (FFFS), bridged by behaviors mediating the conflict between the two (BIS). Further testing involving different species, situations (e.g., in social groups), ages, sexes, and types of rewards should be conducted to confirm the reproducibility of this innovative test and document the multidimensionality of BAS.^16^^,^^64^ The subjects belonged to the same genetic line, were of similar age, originated from the same farm, and underwent similar acclimation and habituation procedures. To ensure generalizability, the STRANGE framework recommends testing the paradigm on more diverse populations.^65^

In the BIBAGO, we used a palatable reward, eaten for pleasure,^66^ and not to alleviate hunger as pigs were fed *ad libitum*, in the presence of a validated negative stimulus,^30^^,^^31^^,^^32^ creating a clear approach-avoidance conflict. Remarkably, this test was the most repeatable among other conventional personality tests and revealed novel aspects of non-human personality that are unambiguously reward related.

Our findings revealed distinct behavior clusters: one comprising chewing, rewards eaten, and interaction with rewards, which we consider as BAS-related behaviors, and vocalizations and freezing, considered as FFFS behaviors. Another cluster comprised interruption of vocalizations and tail wagging behaviors, attributed as BIS behaviors, and human, object, and arena exploratory behaviors, considered as exploration traits. Vocalizations (FFFS) and their interruption (BIS) bridged these clusters. EFA corroborated these findings, demonstrating that BAS and FFFS behaviors have strong interrelationships and are distinguished from BIS behaviors. Consistent with the network approach, vocalizations (FFFS) appear to play a central role bridging BIS and BAS.

Whereas standard BIS/BAS questionnaires consist in self-reporting the level of worry for fictive situations, representing only a putative indicator of behavioral inhibition,^16^^,^^64^ the BIBAGO test does measure consistent reactions to one single approach-avoidance conflict directly. Yet, this single situation was enough to distinguish BIS from BAS as biological systems based on a behavioral approach, as opposed to a lexical approach. For this dichotomy, see the recent revision of the nomenclature referring to the Goal Inhibition and Goal Activation Systems, i.e., GIS/GAS by McNaughton.^67^ This should inspire further *behavioral* studies in other species, including humans, through, e.g., promising testing setups using virtual reality.^68^ The opposing BAS behaviors (interacting with the rewards, eating, and chewing them) and FFFS behaviors (freezing and vocalizing) reflect distinct and conflicting individual motivation strategies: approach vs. avoidance. Conversely, BIS behaviors seem to reflect hesitation when facing uncertainty, as evidenced by vocalization interruptions during both the BIBAGO and NOT. Consistent with this idea, vocalizations measured during the BIBAGO but also during OFT are negatively associated with BIS behaviors. The central role of vocal behaviors in the network warrants further investigations, particularly given the technological advances in bioacoustics, a powerful tool to assess emotional valence.^69^^,^^70^ Accounting for the types of vocalizations such as low-frequency grunts and high-frequency calls (e.g., squeals) that, respectively, indicate positive and negative emotional valence^71^ could help refining the network. For instance, one could expect that positive grunts are in the network more situated next to BAS while less positive vocalizations would be more connected to BIS and FFFS behaviors.

Our results showed an unexpected positive association between BIS and exploration behaviors. One possible explanation is that BIS is typically activated in novelty contexts, and novelty contexts also inherently elicit exploration.^2^ This suggests that BIS is activated during tests like the HAT, NOT, and OFT when exploring novel items, reflecting its role in increasing arousal and orienting in uncertain situations,^20^ since novelty can be both rewarding and threatening.^72^ Interestingly, we demonstrate that novelty triggers reactions that are independent from responses to rewards.

Analogous to the described links between BAS and extraversion in humans,^16^^,^^18^ we did not find a link between BAS and the equivalent construct in animals, sociability,^1^ likely due to our use of food reward rather than social reward. Additionally, a truly equivalent measure to the multifaceted human extraversion is lacking. Extraversion encompasses sociability but also excitement seeking, enthusiasm, or expressiveness.^73^ These facets, though challenging to objectively measure in animals, are crucial for experiencing positive emotions and well-being^74^ and are therefore highly relevant to positive animal welfare.^15^ Our findings provide a foundation for further research on traits related to positive experiences. Further studies using other rewards are needed to better understand the range of positive experiences animals seek and to explore potential BAS facets (e.g., related to social or novel rewards), ultimately improving our understanding of what animals want and like^75^ and promising for treating mental disorders.^76^^,^^77^

The neurophysiological underpinnings of personality have been extensively researched in humans with clear implications in mental health.^11^^,^^12^^,^^13^ Although farm animals personalities have received much attention,^7^ individualized approaches to animal welfare are usually applied to increase farm productivity or for reducing damaging behaviors,^7^^,^^28^^,^^78^^,^^79^ but not on promoting positive mental states. The BIBAGO is a step toward a personalized, positive-based approach to welfare, in line with the current shift toward a focus on positive animal welfare,^15^ i.e., the promotion of positive mental states in animals, instead of the prevention of negative ones.

### Limitations of the study

This study used of a homogeneous population of subjects from the same genetic line and farm and of a similar age. This raises concerns about the generalizability of the findings to more diverse populations.^65^ The study also used only a single type of reward (palatable food), which may have influenced the findings, suggesting that further research with other types of rewards, such as social or novel ones, is needed to fully understand the range of positive experiences animals seek and to explore potential facets of the BAS. The study also acknowledges that a truly equivalent measure to the multifaceted human trait of extraversion is lacking in animals, which prevented a definitive link between BAS and sociability.

## Resource availability

### Lead contact

Requests for further information and resources should be directed to and will be fulfilled by the lead contact, Charlotte Goursot (charlotte.goursot@blv.admin.ch).

### Materials availability

This study did not generate new unique reagents.

### Data and code availability

• Data: All data generated in the study have been deposited at Zenodo: https://doi.org/10.5281/zenodo.15608393 and are publicly available.
• Code: All original code has been deposited at Zenodo: https://doi.org/10.5281/zenodo.15608393 and is publicly available.
• Any additional information required to implement the behavioral test BIS/BAS Goursot (BIBAGO) or reanalyze the data reported in this paper is available from the lead contact upon request.

## Acknowledgments

We acknowledge the colleagues of the Medau farm (Doris, Silvia, Tamara, Dana, and Niko) who helped to take care of the pigs, Jen-Yun Chou who took care of the pigs during the weekends, and Jean-Loup Rault and Océane Schmitt for providing and taking care of the subset of pigs. We thank Christian Haberl very much for his technical support and Jean-Loup Rault for his advice during the design. We thank Alice Balard for creativity support in choosing the term BIBAGO and Sandra Düpjan and Annika Krause for initial support during the conceptualization. We are grateful to all the novel humans for their valuable help: Johanna Neuhauser, Kristina Kull, Jasmin Prise, Nadia Müller, Kimberly Brosche, Luzie Bauer, Helen Zobrist, and Suzanne Truong. This research has been funded by the Deutsche Forschungsgemeinschaft (DFG; GO3558/1-1, MA 9054/1-1). We thank Lisette Leliveld, Conor Goold, and the colleagues during the Journal Club at FBN Dummerstorf for their helpful feedback on previous versions of the manuscript. The publication fees for this article were covered through the financial support of the University of Veterinary Medicine Vienna.

## Author contributions

C.G. conceptualized the original study and acquired funding. C.G., G.F., F.D.A., and S.A. conducted animal testing. C.G., M.C., F.D.A., G.F., and S.A. designed and reported the inter-observer reliability. F.D.A., S.A., and G.F. performed the video analysis. S.C.M.F. performed the statistical analysis. C.G. and S.C.M.F. wrote the manuscript with contribution and feedback from all authors.

## Declaration of interests

The authors declare no competing interests.

## Declaration of generative AI and AI-assisted technologies in the writing process

During the preparation of this work, the authors used Google Gemini in order to improve the language and readability of the paper. After using this tool, the authors reviewed and edited the content as needed and take full responsibility for the content of the publication.

## STAR★Methods

### Key resources table

**Table:** 

REAGENT or RESOURCE	SOURCE	IDENTIFIER
**Deposited data**		

Raw data and code	This paper	https://doi.org/10.5281/zenodo.15608393

**Experimental models: Organisms/strains**		

Domestic pig *(Sus domesticus)*	This paper	N/A

**Software and algorithms**		

R software v. 4.4.0	R core team^80^	https://www.Rproject.org/
mice R package v. 3.16.0	Buuren and Groothuis-Oudshoorn^81^	https://doi.org/10.18637/jss.v045.i03
GUniFrac R package v. 1.8	Chen and Zhang^82^	https://doi.org/10.1093/bioinformatics/btac618
vegan R package v. 2.6–4	Oksanen and colleagues^83^	https://CRAN.R-project.org/package=vegan
rptR R package v. 0.9.22	Stoffel and colleagues^84^	https://doi.org/10.1111/2041-210X.12797
qgraph R package v. 1.9.8	Epskamp and colleagues^85^	https://doi.org/10.18637/jss.v048.i04
igraph R package v. 2.0.3	Csárdi and Nepusz^86^	http://igraph.sf.net
bootnet R package v. 1.6	Epskamp and colleagues^87^	https://doi.org/10.3758/s13428-017-0862-1
psych R package v. 2.4.3	Revelle^88^	https://CRAN.R-project.org/package=psych

**Other**		

Video camera Hikvision DS-2CD5046GO-AP	Hikvision	https://www.hikvision.com/my/products/IP-Products/Network-Cameras/Ultra-Series-SmartIP-/DS-2CD5046G0--AP-/
Video camera HDR-CX900E camcorder	SONY	https://www.sony.co.uk/electronics/handycam-camcorders/hdr-cx900e
Microphone ECM-HGZ1	SONY	https://www.sony.com/electronics/support/product/ecm-hgz1/manuals

### Experimental model and study participant details

The study took place at the Medau pig research and teaching farm of the Vetmeduni Vienna (Berndorf, Austria) and was approved by the Ethics and Animal Welfare Committee of the University of Veterinary medicine, Vienna in accordance with the University’s guidelines for Good Scientific Practice (ETK-175/11/2021) and with the legal requirements of the European Union (directive 2010/63/EU). Piglets were weaned at four weeks of age and a maximum of two siblings per sow were selected. Each subject was randomly given an ID-number, which determined the order of the individual tests throughout the entire experiment. We used a total of 80 healthy uncastrated undocked male piglets (Swiss Large White × Pietrain breed, 5–8 weeks of age), divided into 5 replicates of 16 piglets each. The home pen (7.55 × 2.43 m) contained both slatted floors and a solid concrete section. Access to food and water was provided *ad libitum* while straw, hay and sawdust were given twice daily. For the Novel Peer Test (see personality tests), pigs were given access to an additional pen (6.5 × 2.4 m). Pigs entered this additional pen through a waiting area (2.3 × 2.4 m) that contained a corridor (1.1 × 0.4 m) through which individual pigs had to pass.

From day 4 until 6 after weaning, pigs were habituated to being handled by the experimenters and to treat balls (Interactive Dog Toys - Enrichment IQ Treat Dispenser Ball, Lesfit, Yiwu Baoda Garment Accessories Co., China) containing a mixture of chocolate raisins and salty sticks used in the BIBAGO (“Handling”, 1 h, twice daily, see Figure S1). Most of the general testing procedure has been described in Ambruosi and colleagues.^34^ For each replicate, the experimental period lasted five weeks and consisted of 20 working days. At the end of the experiment, the pigs returned to the regular farm herd.

An additional subset of 24 female piglets (Swiss Large White × Pietrain breed, 5–9 weeks of age) was used. The pigs were used in another experiment (for more details, see the pre-registered study^89^) at different facilities within Medau, Vetmeduni Vienna. This experiment investigated human-pig relationship with pigs randomly assigned to two different treatments that started at 5 weeks of age and assumed to not interfere with the BIBAGO responses: positive (“positive contact”) vs. no human contacts (“control”). Piglets were divided into two replicates of 12 piglets. Sibling pairs were recruited at weaning, with each sibling randomly allocated to a different treatment group. Pigs were housed in groups of three in adjacent pens (2 × 3 m each), making four groups per replicate (2 groups per treatment). Due to illness, 3 pigs from the first replicate were not tested which resulted in a sample size of 21 pigs.

### Method details

#### Testing procedure

The general procedure for the 80 pigs is summarised in Figure S1. During weeks 6 and 8, each piglet was individually subjected to an open field test (OFT, on days 11 and 25), a novel object test (NOT, on days 12 and 26), a human approach test (HAT, on days 13 and 27), a novel peer test (NPT, on days 14 and 28) and the BIS/BAS by Goursot test (BIBAGO, on days 15 and 29). In short, all personality tests (except NPT) were conducted in an arena (2.3 × 2.3 × 1 m). For the OFT and BIBAGO the location of the arena and wall panels were changed so that it looked unfamiliar to the pigs. The OFT lasted for 5 min while the NOT, HAT and BIBAGO each lasted for 6 min (including a minute of habituation to the test arena). During the NOT and HAT, a novel object (spiked rubber toy or construction cone) or an unknown human wearing unusual clothing (thin blue paper overall) was introduced, respectively, into the arena. During the BIBAGO a familiar reward (the treat ball to which the pigs were previously habituated), and a mild negative stimulus, i.e., waving a plastic bag for maximum 3 s, were simultaneously introduced into the arena (see Video S1). The procedure for the NPT is described in Ambruosi and colleagues.^34^ Briefly, the NPT lasted for 6 min and took place in the additional pen that was familiar to the pigs. After 1 min a novel pig was introduced behind a robust, meshed fence (2.5 × 1 m) on the slatted floor area (1.2 × 2.4 m).

Video S1. Procedure of the BIBAGO test for two different individuals, related to STAR Methods: Testing procedure and rationale behind the BIBAGO This video shows the testing procedure of the BIBAGO and focuses on two different pigs showing two contrasting reactions for two behaviors that reflect the behavioral inhibition system (BIS; interruption of vocalisations, i.e., latency to vocalize after the introduction of both stimuli) and the behavioral activation system (BAS; latency to interact with the rewards).

In the additional subset of 24 pigs, the BIBAGO was conducted at 7 and 9 weeks of age (“BIBAGO2”). The experimenter who conducted the tests was blind to the treatments and was unknown by the pigs. During week 5, all pigs were habituated to the treat ball every day (twice a day, five days a week): the experimenter introduced four treat balls filled with salty sticks and chocolate raisins into the home pen containing three pigs. During week 6, the number of sessions was reduced to once per day, three days a week. On the day before each BIBAGO testing (weeks 7 and 9), one habituation session was again conducted to serve as a reminder of the treat ball. This resulted in a total of 15 sessions of 20 min with the treat ball to which each group was exposed to. Although the same procedure for the BIBAGO was followed as for the other pigs, the novel arena dimensions differed (BIBAGO1: 2.04 × 2.20 m; BIBAGO2: 2.20 × 4.95 m).

#### Rationale behind the BIBAGO

We assumed that the duration of interrupting the vocalisations is a BIS-related behavior because it has been previously shown to be combined with an increased arousal and reflect heightened attention in similar test settings.^31^ Moreover, it might resemble “motor planning interruption”, a human BIS item.^16^ Overall, although this behavior has not been pharmacologically validated yet (i.e., anti-anxiety drugs suppress BIS), it could indicate BIS activation, resulting in increased behavioral inhibition, attention and arousal.^22^ The definition of interrupting of vocalisations is only based on the acoustic reaction toward the introduction of stimuli but not on the body posture, hence it differs from the definition of freezing (see Table 1). We assumed that the occurrence and duration of touching the treat ball and eating the rewards would reflect BAS activation. We also recorded the number of vocalisations and the duration of freezing as composite behaviors reflecting both BIS and FFFS, as these behaviors can reflect fear, attention, or avoidance motivations (e.g., flight and freeze are part of FFFS, see Table 1 for references). Based on these assumptions, we reported in Table 1 which variables should be reflected by BIS, FFFS or BAS.

#### Behavioral analyses

For each replicate, two cameras remotely video-recorded the arena test during the personality tests (Hikvision DS-2CD5046GO-AP surveillance cameras and Sony HDR-CX900E camcorder, Sony ECM-HGZ1 microphone). The videos from both cameras were merged using Kdenlive software (version 22.12.2) to combine both video and sound. All recordings were analyzed using the open-source software BORIS version 7.13.6.^90^

For each test, we used a 30-s buffer at the beginning of the video to select a standardised starting point (such as the experimenter away from the camera), resulting in 270 s of test footage analyzed. Out of 800 observations, we excluded 7 OFT observations, 3 HAT observations, 4 NPT and 5 BIBAGO observations due to technical issues. Additionally, the first NPT of the first replicate (16 observations) was excluded from the statistical analyses as it was used as a pilot study to establish the NPT procedure. Hence, 765 observations were included for the statistical analysis (Table S1). During the OFT, NOT, and HAT, the activity, exploration, escaping, and vocal behaviors were observed. The behavioral reactions recorded during the BIBAGO were: the number of interactions with the treat ball and the number of treats eaten, vocalisations (number and duration of interrupting) and freezing duration. During the NPT the location of the pig in the testing pen (front next to the fence, middle, back) and its interactions with the novel pig was recorded. Table 1 shows the ethogram of all recorded behaviors.

Inter-observer reliability training involved two observers at all times. Each session included one experienced ethologist with extensive knowledge in pig behavior and a master’s student undergoing training. The students were trained on an independent video dataset that was not used for the analyses until they demonstrated a high level of agreement with the experienced ethologist (Cohen’s kappa ≥0.70) across multiple pilot sessions. After reaching this threshold, the students completed the behavioral analysis. The intraclass correlation coefficients (ICC) agreement ranged from 0.82 to 1.00, except for freezing (0.67) where the agreement was considered moderate,^91^ whereas the number of observations per test used for the inter-observer reliability ranged from 3 to 16 (see Table S2).

### Quantification and statistical analysis

All data analysis and data handling were conducted in R v. 4.4.0.^80^ Table 1 describes all variables included in the analysis. We had missing observations for the behaviors duration (4.375%) and occurrences of chewing (4.375%), duration (3.125%), latency (3.125%), and occurrences of interacting with the rewards (3.125%), duration (3.125%) and occurrences of freezing (3.125%), duration of interrupting the vocalisations in the BIBAGO (3.125%), number of vocalisations in the BIBAGO (3.125%), number of rewards eaten (5.625%), duration (1.875%), latency (1.875%), and occurrences for exploring the novel human (1.875%), occurrences of jumping during the HAT (1.875%), occurrences of wagging the tail during the HAT (1.875%) for the personality dataset. Multiple imputation was performed with the R package “mice” v. 3.16.0,^81^ using the method “pmm” (predictive mean matching). In addition, all behaviors from the NPT test had 12.5% missing variables due to all animals from one replicate (16 animals) serving as pilot study on time point 1. These values were not imputed, and a reduced dataset is used for further analysis. BIBAGO was additionally tested in an independent group of 21 pigs, 19 of which were tested two times. The variables duration of interrupting the vocalisations and number of vocalisations had 2.5 and 7.5% missing data and were imputed with the same method described above.

In order to reduce redundancy, and ensure reliable, parsimonious results and easier interpretability, we chose to remove variables that have a Pearson’s correlation rho >0.8 in pairwise comparisons. Removed variables were: the occurrences of freezing, chewing, locomotion, walking by the fence, sudden display, being in the back of the pen, being in the middle of the pen; duration of facing the back and being in the front of the pen.

We tested whether the different behavioral reactions as a whole recorded in each test are consistent within individuals between the two timepoints, i.e., the test repeatability. A high repeatability value means individuals are behaving consistently, while a low value means their behavior is less predictable from one time to another. We summarised all behavioral variables for each test into pairwise Aitchison distance matrices to provide an overall repeatability for each test and 1) calculated distance-based ICC (dICC) with the function “dICC” and 1000 iterations of the “GUniFrac” R package v. 1.8,^82^ to quantify how consistently individuals maintained their behavioral profile over time. This method extends the traditional ICC to multivariate data, evaluating how well the distance matrix preserves individual differences across repeated measures; 2) calculated marginal permutational multivariate analysis of variance test (permanova) to assess the marginal contribution of time point (time point 1 vs. time point 2) while controlling for sow and replicate effects as explanatory variables, using the function “adonis2” of the R package “vegan” v. 2.6–4.^83^ This approach tests whether the overall behavioral patterns of the animals changed between the two timepoints. Instead of looking at each behavior separately, it considers all behaviors together to see if, as a group, they differ over time. Repeatability of the NPT test was tested on a reduced dataset (*n* = 64 animals repeated 2 times) due to missing data.

Additionally, we tested the repeatability of each individual behavior within a test with the R package “rptR” v. 0.9.22,^84^ with the Gaussian data type, and 1000 parametric bootstrap iterations. This method is widely used in the personality literature to quantify the proportion of total variation in a behavioral measure that can be explained by consistent differences between individuals, in other words, the repeatability of each single behavior.^29^

To ensure that the links between behavioral variables reflect links between different personality traits, we excluded all behaviors that were not repeatable, i.e., confidence interval includes 0, for further analysis. Excluded variables were latency and occurrences of manipulating the novel object from the NOT test, duration of exploring the human and occurrences of from the HAT test, and latency and occurrences for nose-nose interactions, occurrences for climbing the fence, being in the front of the pen and latency to reach the front of the pen from NPT test, latency to interact with the rewards from the BIBAGO (Table S4). Locomotion, jumping and vocalisations measured during the OFT were not repeatable, but were included in further analysis, as we find it important to compare the OFT with other tests given its widespread use.

We represented the associations between the 20 (17 repeatable and the 3 non-repeatable OFT) behavioral variables from the different tests applying graph theory to construct “psychological networks”, in which a node represents one behavior recorded during a particular test and edges represent associations between these behaviors, after controlling for all other nodes within the network. Network analysis was implemented using Gaussian graphical models (GGMs). This approach has been successfully used in human clinical psychology, psychiatry and personality research (reviewed in^92^^,^^93^), and in animal behavior^94^ and can be used to explore associations between the different behaviors, potentially highlight causal relationships and generate hypotheses on the processes acting on these associations.^95^ For that, we included the behaviors measured at the first exposure (time point 1) which has been suggested more relevant in the context of personality for species habituating quickly to novelty such as pigs.^14^^,^^28^ For the NPT behaviors which did not have time point 1 (first replicate) available, we used the measurements at time point 2. All behaviors were scaled.

For constructing the GGMs, we applied L1 lasso penalties, where the inverse covariance matrix (partial correlations) underwent regularisation through penalised maximum-likelihood estimation.^96^ This regularisation resulted in a sparse graph with non-zero partial correlations considered credible, while partial correlations close to zero were shrunk to zero. To select the optimal tuning parameter, λ value, we employed the Extended Bayesian Information Criterion (EBIC) based on the graph with the lowest EBIC^97^ and implemented it with the R package “qgraph” v. 1.9.8.^85^ EBIC uses a hyperparameter γ that controls how much EBIC prefers a model with fewer edges.^97^ We chose an intermediate γ value of 0.12, that balances the removal of spurious edges and the removal of true edges while prioritising a network with higher sensitivity. The final network was visualised with the R package “igraph” v. 2.0.3.^86^ We applied the walktrap algorithm to identify clusters within the network^98^ implemented in “igraph”, i.e., groups of behaviors (nodes) that are more densely connected with each other than with the rest of the network. To assess the role of individual behaviors (nodes) in the network, we calculated three centrality measures, strength, betweenness and closeness with the function “centralityplot” of the “qgraph” R package. Strength measures how strongly a node is directly connected to other behaviors in the network. A node with high strength influences or is influenced by many others. Betweenness indicates how often a node lies on the shortest path between two other nodes. A node with high betweenness may act as a “bridge” or mediator between different nodes or clusters. Closeness reflects how quickly a behavior can reach all other behaviors in the network. Higher closeness means a node is more centrally positioned and can potentially influence others more efficiently. To evaluate the stability of these centrality measures, we used the function “bootnet” of the R package “bootnet” v. 1.6.^87^

Complementary to the network approach, we further investigated the connection of the motivational systems and personality traits with extended exploratory factor analysis (EFA) with the Dwyer’s factor extension^99^ implemented in the “psych” package v. 2.4.3.^88^ All behaviors were scaled, and a correlation matrix was created with Pearson correlation. Sampling adequacy was assessed with the Bartlett sphericity test and the Kaiser-Meyer-Olkin (KMO) factor adequacy for each behavior. The functions “cortest.bartlett” and KMO were used and based on the measure of sampling adequacy (MSA) values. The number of factors was estimated with scree plots within parallel analysis, with the function “fa.parallel”. The exploratory factor analysis was implemented with 2 factors, oblimin rotation and a principal factor solution with the functions “fa” and “fa.extension”. The relations between the resulting factors and the behaviors are considered interpretable if factor loadings are minimum 0.3.^100^ Factor scores for each individual were calculated based on regression-based weights with the function factor.scores and adequacy was evaluated with indices of determinacy.
