# Supplementary material for: Bridging human and animal personality: A behavioral test to assess reward sensitivity
Source: iScience. 2025 Sep 2;28(10):113487. doi: 10.1016/j.isci.2025.113487 (PMC12513275; doi:10.1016/j.isci.2025.113487)
Supplement: Document S1. Figures S1 and S2 and Tables S1–S6 [file mmc1.pdf]

iScience, Volume *28*

Supplemental information

Bridging human and animal personality:

A behavioral test to assess reward sensitivity

Susana C.M. Ferreira, Fabiana De Angelis, Sarah Ambruosi, Giulia Ferroni, Matteo Chincarini, and Charlotte Goursot

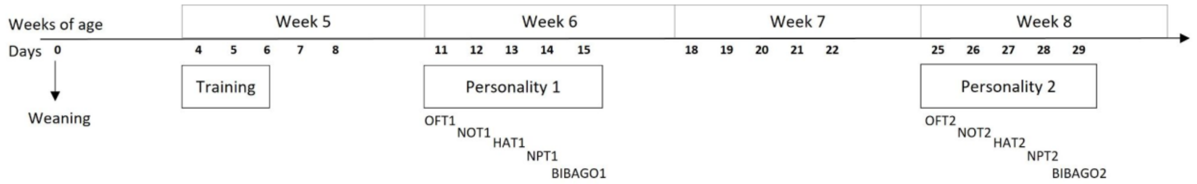

Figure S1. General experimental procedure for each replicate, Related to STAR Methods: Testing procedure. The numbers in the bottom row indicate the days after weaning, whereas the numbers in the top row indicate the age in weeks. Pigs were not tested during the weekends (days 9, 10, 16, 17, 23, 24, 30, 31). OFT: Open Field Test; NOT: Novel Object Test; HAT: Human Approach Test; NPT: Novel Peer Test; BIBAGO: BIS/BAS Goursot test. NOT1: spiked rubber toy as novel object; NOT2: construction cone as novel object.

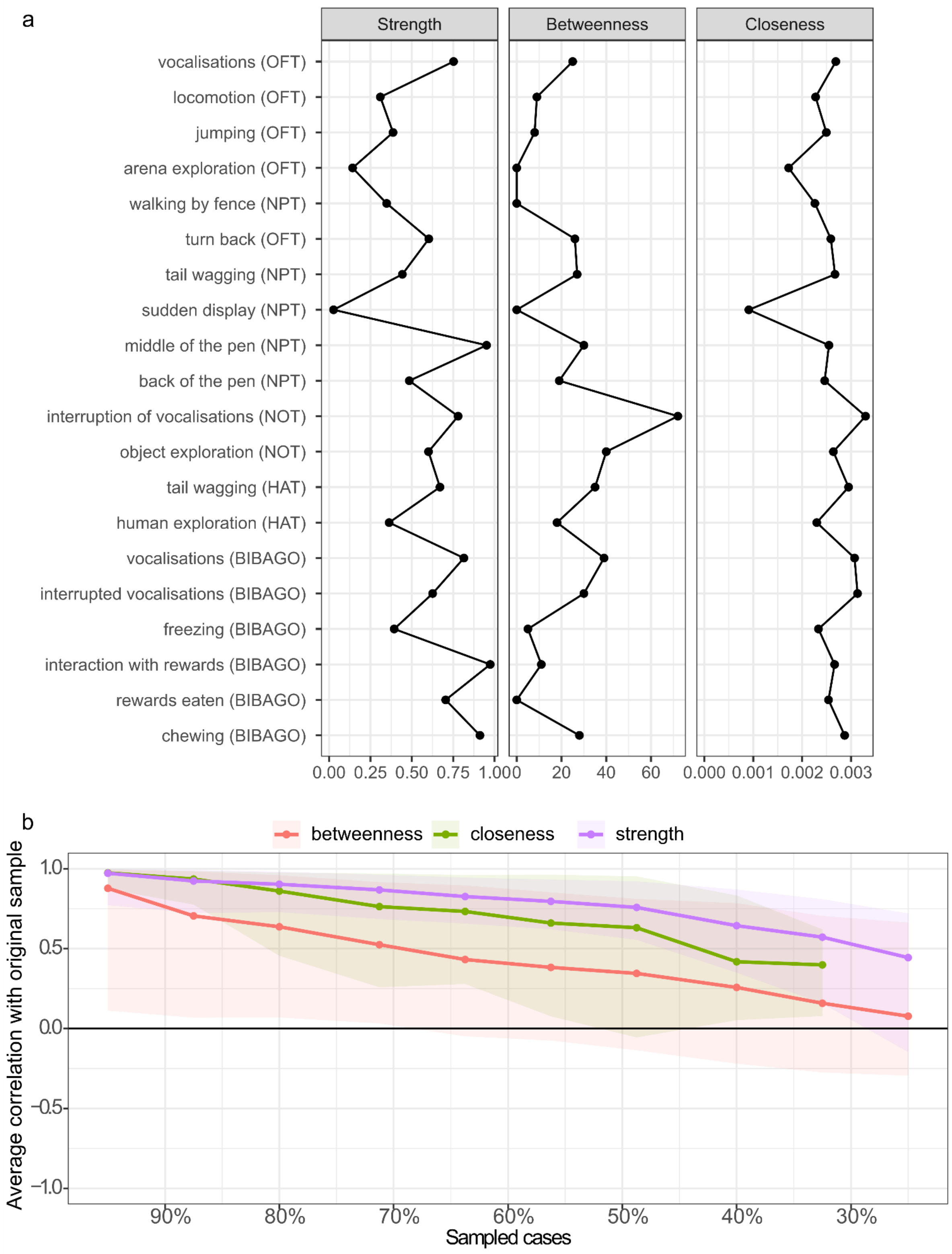

Figure S2 Network centrality, related to Figure 2. a) Observed strength, betweenness and closeness centrality values b) Stability of strength, betweenness and closeness centrality values and respective confidence intervals. Correlation-stability coefficients of centrality values were calculated with case-dropping bootstrap.

Table S1. Number of observations excluded in the study for each test, related to STAR Methods: Behavioural analyses

Type of test	# of subjects included	# of subjects excluded	Reasons for excluding
OFT	160 observations<br>= 80 OFT1; 80 OFT2<br>= 80 subjects with both observations	0 observations	-
NOT	160 observations<br>= 80 NOT1; 80 NOT2<br>= 80 subjects with both observations	0 observations	-
HAT	157 observations<br>= 80 HAT1; 77 HAT2<br>= 77 subjects with both observations	3 observations<br>= 0 HAT1; 3 HAT2<br>= 3 subjects	Sound disturbance bout (40s, 70s, 100s)
NPT	144 observations<br>= 64 NPT1; 80 NPT2<br>= 64 subjects with both observations	16 observations<br>= 16 NPT1; 0NPT2<br>= 16 subjects	Batch 1 NPT1 was used to develop NPT
BIBAGO	155 observations<br>= 77 BIBAGO1; 78 BIGAGO2<br>= 75 subjects with both observations	5 observations<br>= 3 BIBAGO1; 2 BIBAGO2<br>= 5 subjects	Treatball opened unintentionally while being placed in the arena by the experimenter

Table S2. Inter-observer reliability for each behaviour during personality testing, using the intraclass correlation coefficient ($ICC_{agreement}$), related to STAR Methods: Behavioural analyses. (1) The behaviour was rare and therefore the values are not statistically meaningful. N: number, D: duration, L: latency.

Behaviour	Test	$ICC_{agreement}$	$CI_{95\%}$	Total observations
Arena exploration (D)	OFT	0.826	[0.432; 0.954]	10
Locomotion (D)	OFT	0.959	[0.552; 0.993]	8
Vocalisations (N)	OFT	0.979	[0.909; 0.996]	8
Jumping (N)	OFT	1[(1)]	[1; 1][(1)]	8
	HAT	1[(1)]	[1; 1][(1)]	6
Object exploration (D)	NOT	0.996	[0.946;1]	4
Object exploration (N)	NOT	0.839	[-0.041; 0.989]	4
Object exploration (L)	NOT	0.990	[0.874; 0.999]	4
Interruption of vocalisations (D)	NOT/BIBAGO	0.998	[0.990; 1.000]	8
Human exploration (D)	HAT	0.976	[0.893; 0.995]	8
Human exploration (L)	HAT	1	[1; 1]	8
Back of the pen (D)	NPT	0.998	[0.956; 1.000]	3
Front of the pen (N)	NPT	1	[1; 1]	6
Middle of the pen (D)	NPT	0.999	[0.996; 1.000]	6
Turning back (N)	NPT	1[(1)]	[1; 1][(1)]	6
Walking by the fence (D)	NPT	0.890	[0.405; 0.984]	6
Sudden display (D)	NPT	0.891	[0,491; 0,984]	6
Tail wagging (N)	NPT	0.970	[0.886; 0.992]	11
Nose-nose interactions (L)	NPT	1.000	[0.999; 1.000]	5
Nose-nose interactions (N)	NPT	0.908	[0.478; 0.987]	6
Vocalisations (N)	BIBAGO	0.979	[0.909; 0.996]	8
Interruption of vocalisations (D)	BIBAGO	0.995	[0.985; 0.998]	16
Chewing (D)	BIBAGO	0.949	[0.818; 0.986]	12
Interactions with reward (D)	BIBAGO	0.987	[0.964; 0.995]	16
Interactions with reward (L)	BIBAGO	1	[1; 1]	16
Freezing (D)	BIBAGO	0.672	[0.299; 0.870]	16

Table S3. Marginal permutational multivariate analysis of variance (permanova) testing the effects of time point, controlling for mother ID and replicate effects for all the behaviours within each personality test. Related to Results: Open field test has the lowest repeatability across time.

Test		Time point	Mother ID	Replicate	Residual	Total
BIBAGO	df	1	40	2	114	159
	R2	0.034	0.326	0.009	0.559	1.00
	p	0.001	0.002	0.446	-	-
BIBAGO2	df	1	1	1	36	39
	R2	0.051	0.082	0.006	0.835	1.00
	p	0.017	0.030	0.735	-	-
NOT	df	1	40	2	114	159
	R2	0.026	0.282	0.005	0.632	1.00
	p	0.004	0.052	0.822	-	-
HAT	df	1	40	2	114	159
	R2	0.074	0.226	0.008	0.614	1.00
	p	0.001	0.382	0.563	-	-
OFT	df	1	40	2	114	159
	R2	0.237	0.271	0.007	0.469	1.00
	p	0.001	0.001	0.471	-	-
NPT	df	1	40	2	94	139
	R2	0.016	0.340	0.021	0.570	1.00
	p	0.005	0.017	0.088	-	-

Table S4. Repeatability for each behaviour, related to Results: Open field test has the lowest repeatability across time. Coloured shades highlight the behaviours that were removed for further analysis due to low repeatability. Note: all behaviours measured during OFT were included in further analysis, as we find it important to compare the OFT with other tests, given its widespread use.

Name	Test	R	CI	P
Arena exploration (D)	OFT	0.360	[0.148, 0.535]	<0.001
Locomotion (D)	OFT	0.007	[0.000, 0.231]	0.499
Vocalisations (N)	OFT	0.000	[0.000, 0.211]	0.5
	BIBAGO	0.418	[0.225, 0.590]	<0.001
	BIBAGO2	0.671	[0.351, 0.864]	0.003
Jumping (N)	OFT	0.000	[0.000, 0.213]	0.5
	HAT	0.013	[0.000, 0.220]	0.474
Object exploration (D)	NOT	0.472	[0.290, 0.622]	<0.001
Object exploration (N)	NOT	0.136	[0, 0.343]	0.122
Object exploration (L)	NOT	0.000	[0.000, 0.213]	1
Interruption of vocalisations (D)	NOT	0.491	[0.315, 0.649]	<0.001
			[0.081, 0.472]	0.005
	BIBAGO	0.289	[0, 0.699]	0.042
	BIBAGO2	0.402		
Human exploration (L)	HAT	0.248	[0.032, 0.434]	0.014
Human exploration (D)	HAT	0.000	[0.000, 0.223]	1
Tail wagging (N)	HAT/	0.391/	[0.177, 0.562]	<0.001
			[0.171, 0.586]	<0.001
	NPT	0.397		
Chewing (D)	BIBAGO	0.322	[0.116, 0.505]	0.002
Rewards eaten	BIBAGO/	0.458	[0.279, 0.615]	<0.001
	BIBAGO2	0.527	[0.147, 0.785]	0.0163
Interactions with reward (D)	BIBAGO/	0.508	[0.333, 0.658]	<0.001
	BIBAGO2	0.377	[0, 0.695]	0.0495
Interactions with reward (L)	BIBAGO/	0.05/	[0, 0.259]	0.347
	BIBAGO2	0	[0, 0.438]	1
Freezing (D)	BIBAGO/	0.442/	[0.254, 0.593]	<0.001
	BIBAGO2	0.559	[0.204, 0.801]	0.003
Back of the pen (D)	NPT	0.544	[0.352, 0.689]	<0.001
Climbing the fence (N)	NPT	0.125	[0, 0.363]	0.145
Front of the pen (N)	NPT	0.241	[0, 0.463]	0.0325
Front of the pen (L)	NPT	0.007	[0, 0.253]	0.499
Middle of the pen (D)	NPT	0.633	[0.472, 0.767]	<0.001
Sudden display (D)	NPT	0.392	[0.165, 0.586]	<0.001
Turning back (N)	NPT	0.388	[0.153, 0.575]	<0.001
Walking by the fence (D)	NPT	0.523	[0.318, 0.69]	<0.001
Nose-nose interactions (L)	NPT	0	[0, 0.252]	0.5
Nose-nose interactions (N)	NPT	0.084	[0, 0.347]	0.267

Table S5. Factor analysis of behaviours reflecting approach-avoidance motivations: behavioural inhibition (BIS), behavioural activation (BAS), fight-flight-freeze systems (FFFS). Related to Figure 3. Shown are the factors (PA1 and PA2). BIBAGO: BIS/BAS Test, NOT: Novel Object Test, HAT: Human Approach Test, NPT: Novel Peer Test.

Behaviour	PA1	PA2
chewing (BIBAGO)	0.887	
interaction with rewards (BIBAGO)	0.904	
freezing (BIBAGO)	-0.391	-0.187
interruption of vocalisations (BIBAGO)		0.736
vocalisations (BIBAGO)	-0.542	-0.402
rewards eaten (BIBAGO)	0.779	
interruption of vocalisations (NOT)		0.712
tail wagging (HAT)		0.609
tail wagging (NPT)		0.262
**SS loadings**	2.673	1.690
**Proportion variance**	0.297	0.188
**Cumulative variance**	0.297	0.485

Table S6. Extended factor analysis of behaviours reflecting approach-avoidance motivations (BIS, BAS, FFFS) to behaviours reflecting classic personality traits (sociability, exploration and unknown). Related to Figure 3. Shown are the factors (PA1 and PA2), the sum of the squared loadings, i.e. the communality (h2) and the residual variance, i.e. the uniqueness (u2). BIS: behavioural inhibition; BAS: behavioural activation; FFFS: fight-flight- freeze systems (FFFS). NOT: Novel Object Test, OFT: Open Field Test, HAT: Human Approach Test, NPT: Novel Peer Test.

Behaviour	PA1	PA2	h2	u2
object exploration (NOT)	-0.079	0.457	0.2079	0.792
jumping (OFT)	-0.175	-0.175	0.0675	0.933
locomotion (OFT)	0.242	0.085	0.0701	0.93
vocalisations (OFT)	0.124	-0.455	0.2104	0.79
object exploration (OFT)	-0.094	0.319	0.1043	0.896
human exploration (HAT)	0.06	-0.323	0.1038	0.896
back of the pen (NPT)	0.203	-0.075	0.0436	0.956
middle of the pen (NPT)	0.019	0.12	0.0152	0.985
turning back (NPT)	-0.147	0.118	0.032	0.968
walking by fence (NPT)	-0.161	-0.029	0.0276	0.972
SS loadings	0.200	0.683	-	-
Proportion variance	0.020	0.068	-	-
Cumulative variance	0.020	0.088	-	-
Proportion explained	0.226	0.774	-	-
Cumulative proportion	0.226	1.000	-	-
